# Identification of the Key Aroma Compounds in Condensed Hardwood Smoke

**DOI:** 10.3390/molecules30030720

**Published:** 2025-02-05

**Authors:** Timothy Vazquez, Edisson Tello, Devin G. Peterson

**Affiliations:** Department of Food Science and Technology, Parker Food Science & Technology Building, The Ohio State University, 2015 Fyffe Rd., Columbus, OH 43210, USA; vazquez.115@osu.edu (T.V.); tellocamacho.1@osu.edu (E.T.)

**Keywords:** liquid smoke, condensed hardwood, aroma analysis

## Abstract

The aroma composition of condensed hardwood smoke generated from a mixed hardwood was characterized by gas chromatography–mass spectrometry/olfactometry analysis. Twenty-seven odorants with a flavor dilution value ≥4 were identified and quantified, revealing 19 compounds reported with an odor activity value >1 at a 0.3% dosage level. The odorants with the highest dilution values were 2,3-butanedione (buttery), guaiacol (clove, vanilla), 4-methylguaiacol (toasted, vanilla, ashy), 3-ethylphenol (ashy), 4-methylsyringol (burnt, plastic, clove), and butyric acid (cheesy). Sensory descriptive analysis revealed that the condensed smoke consisted of eight main attributes, namely ashy, burnt–sulfurous, creosote, green–woody, pungent, smoky, spicy–sweet, and woody. No significant differences in the aroma attributes were reported between the condensed smoke and the corresponding recombination model, indicating that the odorants effectively captured the characteristic aroma of the condensed smoke.

## 1. Introduction

The smoking of foods has been in practice for thousands of years to increase their shelf life and enhance the flavor in a wide variety of different products. The functionality of smoke has been attributed to the complex composition of chemical compounds that adsorb and react with food during the smoking process. Conventional smoke is an aerosol that forms from the pyrolysis of wood—usually hardwood [1]. Most pyrolysis occurs between 200 and 500 °C, which is attributed to the thermal degradation temperatures of the main components of hardwoods, specifically cellulose, hemicellulose, and lignin [2]. Cellulose and hemicellulose form the majority of organic acids, linear and cyclic ketones, aldehydes, and furans, similar to products formed by heating sugar and starches [3,4,5]. On the other hand, lignin pyrolysis generates primarily phenolic compound derivates [6]. In addition, pyrolysis generates numerous other large organic molecules, including anhydrosugars, oligomers, and hydrocarbons, which comprise the complex tar phase [7,8].

Conventionally, smoke is generated within or near the smokehouse under conditions that pose challenges for consistent application. Consequently, manufacturers more commonly rely on smoke that has been condensed, known as condensed smoke or liquid smoke, which allows for more control and consistency [9,10]. Condensed smoke is frequently used to study smoke’s composition due to the ease of extraction, fractionation, and the addition of analytical standards [1,11].

Condensed smoke has been characterized as containing different chemical classes of compounds. Notably, the phenolic fractions within it exhibit the most pronounced aroma intensity [1]. Phenolic compounds like guaiacol, 4-methylguaiacol, and syringol are recognized as significant contributors to the aroma and flavor of smoke, although other phenolics, such as *o*-cresol, *p*-cresol, and 4-ethylguaiacol, have been demonstrated to influence both the intensity and character of the smoke [12]. Further studies on condensed smoke have enabled the identification of over 400 volatile compounds in smoke materials [10,13,14,15]. However, a comprehensive understanding of the specific compounds that contribute to the aroma characteristics of smoke materials remains lacking. The aromas of numerous smoked foods, including pork loin [16], fermented sausages [17], cheese [18], fish [12], and cured pork [19], have been investigated. However, the origin of the aroma compounds cannot be conclusively attributed to the smoke components rather than the food products themselves.

The objective of the present study was to identify the compounds that contribute to condensed hardwood’s smoke aroma. Condensed smoke was obtained from a pilot-scale rapid thermal pyrolysis generator and analyzed by aroma extract dilution analysis (AEDA). The identification of aroma-active components in condensed smoke will enhance the understanding of the smoke flavor in finished products.

## 2. Results and Discussion

### 2.1. Identification of Aroma-Active Compounds

A total of thirty-nine odor-active regions were initially detected in the condensed smoke by GC-MS/O analysis, of which twenty-four were selected for further analysis based on a flavor dilution (FD) factor cut-off value of ≥4, which are shown in Table 1. The most frequent odor descriptors were buttery, floral, sweet–smoky, phenolic, and ashy. Subsequent mass spectrometry analysis identified most of the compounds associated with the odor-active region, with their identities confirmed using authentic standards. However, three odor regions were reported to contain co-eluting phenolic isomers. These isomers were not differentiated during GC-MS/O analysis on the DB-5 column due to similar linear retention index (LRI) values and odor characteristics and similar mass fragmentation patterns (Table 1). The phenolic compounds were successfully separated using a DB-WAX column, leading to the identification of three additional compounds and a final total of twenty-seven odorants.

All compounds identified (Table 1) have been previously reported as chemical components of wood smoke. However, in comparison to previous studies that conducted GC-O analysis on smoke or smoked foods [1,9,10,14,15,20,21], the current study revealed ten additional compounds detected as odorants, which included acetol, 2-methyl-2-cyclopentenone, acetoxyacetone, 3-methyl-2(5H)-furanone, 2,6-dimethylphenol, maltol, 2-ethylphenol, 2,5-dimethylphenol, 2,4-dimethylphenol, 3,4-dimethylphenol, and acetovanillone. Some of these odorant compounds have been reported in other samples, such as caramel [3], toasted rice [4], char-barrel-aged whisky [22], and vanilla [23].

In review, the smoke odorants detected comprised aldehydes, ketones, lactones, acids, and phenolics, which arise from the thermal breakdown of cellulose, hemicellulose, and lignin [24].

**Table 1:** Aroma compounds in condensed smoke with flavor dilution factor of ≥4.

Compound	Aroma Descriptor	LRI		Flavor Dilution Factor	Smoke Concentration (μg/g ± SD)	Typical Application Concentration at 0.3% Dosage (μg/g) ^a^	Odor Threshold in Water (μg/g)	Theoretical OAV ^b^ (0.3% Dosage)
		DB-5	DB-WAX					
2,3-Butanedione	buttery	595	977	64	1377 ± 130	4.20 ± 0.39	0.004 ^d^	1050
Acetic acid	vinegar	653	1442	4	273,890 ± 18,513	821.7 ± 55.5	22 ^f^	37
Acetol	sweet caramel	690	1315	32	92,776 ± 5559	278.4 ± 16.8	100 ^e^	3
2,3-Pentanedione	toasted, buttery, caramel	708	1055	8	159 ± 18	0.48 ± 0.05	0.02 ^e^	24
Butyric acid	cheesy	805	1661	32	837 ± 76	2.52 ± 0.23	0.05 ^f^	50
Furfural	nutty, brothy, caramel	831	1477	16	31,650 ± 1605	95.1 ± 4.8	3 ^e^	32
Acetoxyacetone	sour, dairy	862	1466	64	2204 ± 135	6.60 ± 0.39	20 ^c^	<1
2-Methyl-2-cyclopentenone	floral, fruity, medicinal	905	1394	8	823 ± 62	2.46 ± 0.19	4.4 ^c^	<1
2-Acetylfuran	buttery, sweet	910	1523	16	362 ± 29	1.08 ± 0.09	10 ^f^	<1
3-Methyl-2(5H)-furanone	green, woody, soap	972	1750	8	1161 ± 73	3.48 ± 0.22	3.1 ^c^	1.1
*o*-Cresol	green, pine, phenolic	1051	2012	4	1063 ± 74	3.30 ± 0.22	0.65 ^f^	5
*p*-Cresol	burnt, plastic, clove	1072 *	2094	32	869 ± 47	2.61 ± 0.14	55 ^f^	<1
*m*-Cresol	burnt, plastic, clove	1072 *	2103	32	466 ± 30	1.41 ± 0.09	0.19 ^f^	7
Guaiacol	clove, vanilla	1088	1880	128	2652 ± 113	8.10 ± 0.33	0.012 ^d^	675
2,6-Dimethylphenol	burnt, phenolic, ashy	1108	1923	4	119 ± 8	0.36 ± 0.02	0.4 ^g^	<1
Maltol	sweet, cooked sugar	1111	2027	32	1139 ± 82	3.30 ± 0.25	2.5 ^e^	1.3
2-Ethylphenol	ashy	1134	2078	4	85 ± 5	0.26 ± 0.02	0.3 ^i^	<1
2,5-Dimethylphenol	sweet burnt	1146	2088	4	32 ± 4	0.10 ± 0.01	0.4 ^g^	<1
2,4-Dimethylphenol	burnt, smoky	1154	2090	8	692 ± 22	2.07 ± 0.07	0.5 ^j^	4
4-Ethylphenol	ashy	1163 *	2187	32	226 ± 17	0.69 ± 0.05	0.021 ^j^	33
3-Ethylphenol	ashy	1164 *	2195	32	76 ± 7	0.23 ± 0.020	0.0017 ^j^	134
4-Methylguaiacol	toasted, vanilla, ashy	1191 *	1977	32	1481 ± 79	4.50 ± 0.24	0.021 ^f^	214
3,4-Dimethylphenol	sweet, ashy	1191 *	2235	32	59 ± 5	0.18 ± 0.01	1.2 ^g^	<1
4-Ethylguaiacol	smoky, creosote	1277	2050	8	582 ± 42	1.74 ± 0.13	0.05 ^h^	35
Syringol	ashy, smoky	1349	2283	4	11,911 ± 575	35.70 ± 1.71	1.85 ^f^	19
4-Methylsyringol	woody, vanilla, ashy	1443	2369	64	3353 ± 71	10.20 ± 0.21	0.22 ^c^	46
Acetovanillone	sweet, vanilla	1487	2677	16	2248 ± 148	6.60 ± 0.45	1 ^k^	7

^a^—0.3% application rate represents a reasonable usage level in a final application such as brine, soup, or sauce; ^b^—OAV is calculated by dividing the concentration by the odor threshold; ^c^—threshold level was determined experimentally; ^d^—Rychlik et al., 1998 [25]; ^e^—Buttery et al., 1999 [4]; ^f^—Leffingwell and associates [26]; ^g^—Fenaroli 2010 [27]; ^h^—Van Gemert 2011 [28]; ^i^—Pang et al., 2019 [29]; ^j^—Czerny 2008 [30]; ^k^—Marcq and Schieberle 2015 [31]; * co-eluting compounds on DB-5 columns in detected odor-active regions.

### 2.2. Quantification of Odorants

The concentrations of the twenty-seven odorants ranged from 32 µg/g for 2,5 dimethylphenol to 270,000 μg/g for acetic acid (Table 1). Following acetic acid, the most prevalent compounds were acetol (92,000 µg/g), furfural (31,000 µg/g), and syringol (12,000 µg/g). Considering that the standard dosage of condensed smoke in a finished food product, like soup or brine, is roughly 0.3% *w*/*w* [32,33,34,35,36,37,38,39,40,41], the concentration range at this dosage would fall between 0.0017 and 800 µg/g. The overall compound composition was in general agreement with those reported in smoke food products [10], with the notable exception of higher quantities of low-molecular-weight carbonyl compounds like 2,3-butanedione, 2,3-pentanedione, and 2-methyl-2-cyclopentenone, along with increased levels of phenolics such as acetovanillone. Condensed smoke ingredients typically undergo different processing steps—for example, concentration under a vacuum—which could result in the loss of low-molecular-weight compounds and explain some of the noted differences observed in the chemical composition with prior findings [10]. Furthermore, variations in the wood type utilized or the temperature of the thermal treatment for smoke production would also be expected to influence the smoke composition.

### 2.3. Formation of Aroma-Active Compounds from Hardwood

Phenolic compounds are formed from the pyrolysis of lignin, which consists of three types of phenylpropane monomer units, guaiacyl, syringol, and hydroxyphenyl [42]. In hardwood, monomer lignin units are primarily connected by β-ether linkages in the *para*-position to the hydroxyl group, as well as α-ether, β-aryl, and biphenyl linkages [6,43]. In general, ether linkages are the most susceptible to thermal degradation and can form into a variety of side chains, such as the ethyl ketone found in acetovanillone (2248 μg/g) and the alkyl substitutions found in 4-methylguaiacol (1481 μg/g), 4-ethylguaiacol (1277 μg/g), 4-methylsyringol (3353 μg/g), 4-ethylphenol (226 μg/g), and *p*-cresol (869 μg/g) [6]. Further removal of the side chain results in the formation of guaiacol (2652 μg/g) and syringol (11,911 μg/g) [6].

During pyrolysis, a secondary reaction converts the methoxy groups of guaiacol and syringol into methyl groups through a quinone methide intermediate [6]. This reaction more favorably occurs in the ortho-position and is responsible for the higher abundance of methyl substitutions in the ortho-position found in *o*-cresol (1063 μg/g), 2,6-dimethylphenol (119 μg/g), and 2,4-dimethylphenol (692 μg/g), as opposed to the meta-position substitutions to form *m*-cresol (466 μg/g), 3-ethyphenol (76 μg/g), and 2,5-dimethylphenol (32 μg/g).

The formation of carbonyl compounds in condensed smoke is primarily attributed to the carbohydrates present in the cellulose and hemicellulose of wood cell walls. The degradation of carbohydrates is evident through the dominant presence of sugar degradation products, including acetol (92,776 μg/g) and acetoxyacetone (2204 μg/g) [44], as well as acetic (273,890 μg/g) and butyric acid (837 μg/g) [45]. Furthermore, 2,3-butanedione (1377 μg/g) and 2,3-pentanedione (159 μg/g) can be formed through the reaction of sugar degradation products like hydroxypropanone or hydroxybutanone with formaldehyde [46]. Cyclic compounds such as furfural (31,650 μg/g), 2-acetylfuran (362 μg/g), and 3-methyl-2-(5H)-furanone (1161 μg/g), as well as maltol (1139 μg/g) and 2-methyl-2-cyclopentenone (823 μg/g), can be formed from the thermal dehydration of sugars during pyrolysis [44].

### 2.4. Determination of Odor Thresholds and Calculation of a Theoretical OAV

Odor threshold values are used in combination with compound concentrations to determine the odor activity value (OAV) to estimate odorants’ contributions to the aromas of food products [47]. The odor thresholds in water for the twenty-seven compounds are reported in Table 1; four were experimentally determined, namely 2-methyl-2-cyclopentenone (4.4 μg/g), 3-methyl-2(5H)-furanone (3.1 μg/g), 4-methyl-syringol (0.22 μg/g), and acetovanillone (1.0 μg/g). Based on a typical food application usage level (0.3% *w*/*w*), the corresponding concentrations of the twenty-seven odorants were compared with the corresponding threshold values, and this revealed that 2,3-butanedione (buttery), guaiacol (clove, vanilla), 4-methylguaiacol (toasted, vanilla, ashy), and 3-ethylphenol (ashy) had the highest OAVs, followed by 4-methylsyringol (burnt, plastic, clove) and butyric acid (cheesy). Therefore, these compounds were suggested to have a high contribution to the aroma profile of condensed smoke or aqueous smoke flavorings in food products. With the exception of 3-ethylphenol, these compounds are all frequently reported with high OAVs in other foods, including coffee [48,49], cheese [47,50], and meats [16,17]. Among the twenty-seven compounds, eight compounds (2-methyl-2-cyclopentenone, acetoxyacetone, 2-acetylfuran, 2,6-dimethylphenol, 2-ethylphenol, 2,5-dimethylphenol, p-cresol, and 3,4-dimethylphenol) were reported to be below the OAV, suggesting a negligible contribution to the aroma profile.

Interestingly, 3-ethylphenol (ashy) has only been reported once in hardwood smoke [10] or other smoked foods [17]. The low concentration of 3-ethylphenol (76 μg/g), the co-elution with the more abundant compound 4-ethylphenol (226 μg/g) in the DB-5 column, and the nearly identical MS fractionation pattern likely contributed to the low detection frequency in the prior literature [10,17].

### 2.5. Descriptive Analysis

**Figure 1:**
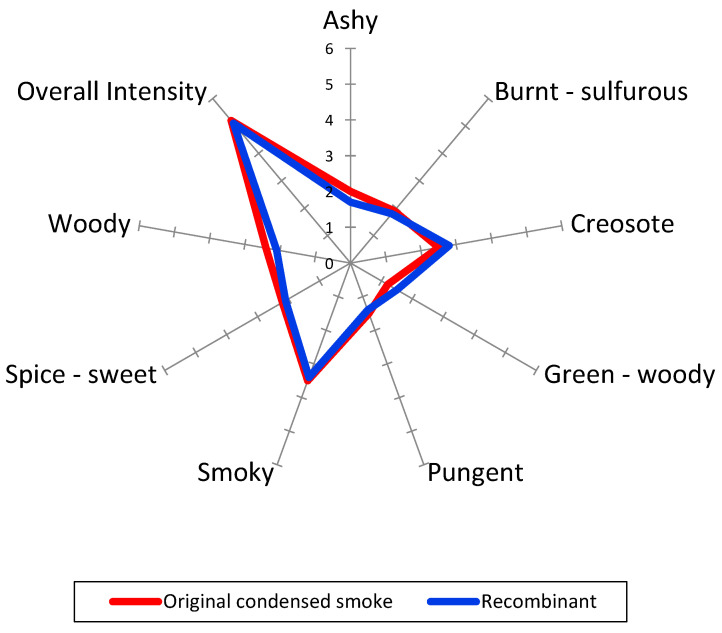
Sensory profile of condensed smoke and recombination model (N = 7, in duplicate). No significant differences were found between samples for all attributes (α = 0.05, linear discrimination model with replicate and panelist interactions).

The sensory descriptive analysis revealed that the condensed smoke consisted of eight main attributes (ashy, burnt–sulfurous, creosote, green–woody, pungent, smoky, spicy–sweet, woody), which are illustrated in Figure 1, along with the comparative analysis of the aroma recombination model. No significant differences were reported in all eight attributes between the samples (α = 0.05), indicating that the condensed smoke was adequately characterized by the 27 compounds included in the recombinant model. Replicates and the panelist by sample interactions were not significant, indicating good reproducibility and attribute concept alignment (Table 2).

The aroma attributes of phenolic compounds are known to be complex. In model smoke systems, the synthetic integration of the odorants has been reported to contribute to new attributes of the overall smoke aroma [51,52]. In the current study, a detailed examination of the odor properties of each compound revealed discernible patterns. Methoxylated phenolic compounds such as guaiacol (clove, vanilla), 4-methylguaiacol (woody, vanilla, ashy), 4-ethylguaiacol (smoky, creosote), syringol (ashy, smoky), and 4-methylsyringol (woody, vanilla, ashy) likely contribute significantly to the smoky character. Alkyl phenols, including 3-ethylphenol (ashy), 4-ethylphenol (ashy), 2,4-dimethylphenol (burnt, smoky), and m-cresol (burnt, plastic, clove), may impart burnt–sulfurous, ashy, and creosote notes. The distinctive green woody and woody aromas can likely be attributed to various compound classes, including 2-methyl-2-cyclopentenone (floral, fruity, medicinal), 3-methyl-2(5H)-furanone (green, woody, soapy), o-cresol (green, pine, phenolic), and 4-methyl-syringol (woody, vanilla, ashy). Similarly, the spicy–sweet aroma likely resulted from a composite mixture of compound classes contributing to the sweet characteristics associated with brown spices and brown sugar, including furfural (nutty, brothy, caramel), 2,3-butanedione (buttery), 2,3-pentanedione (toasted, buttery, caramel), maltol (cooked sugar, sweet), guaiacol (clove, vanilla), and acetovanillone (sweet, vanilla). Furthermore, the pungency of the samples is likely enhanced by acids, particularly acetic acid.

In conclusion, 27 odorants were identified as contributors to the aroma characteristics of condensed hardwood smoke. Gaining a deeper understanding of the smoke flavoring components provides opportunities to improve the quality of smoke-flavored ingredients and products.

## 3. Materials and Methods

### 3.1. Condensed Smoke Samples

A condensed smoke sample was generated by Red Arrow Products (Manitowoc, WI, USA). A mixture of hardwood sawdust was pyrolyzed on a lab-scale rapid thermal pyrolysis smoke generator. The resulting condensed smoke, obtained without additional dilution or processing, was in the form of an aqueous pyrolysis product with a concentration of 70° Brix and a pH of 3.4. Subsequently, the samples were preserved at 4 °C in opaque high-density polyethylene bottles until analysis.

### 3.2. Chemicals

Compounds 2,3-butanedione, 2,3-pentanedione, hydroxyacetone, 2-methyl-cyclopentenone, acetic acid, 1-acetoxyacetone, 2-furaldehyde (furfural), 2-acetylfuran, butyric acid, 3-methyl-2(5H)-furanone, 2-methoxyphenol (guaiacol), 2,6-dimethylphenol, 2-methoxy-4-methylphenol (creosol), maltol, 2-methylphenol (o-cresol), 2-methoxy-4-ethylphenol, 2-ethylphenol, 2,5-dimethylphenol, 2,4-dimethylphenol, 4-methylphenol (p-cresol), 3-methylphenol (m-cresol), 4-ethylphenol, 3-ethylphenol, 3,4-dimethylphenol, 2,6-dimethoxyphenol (syringol), 3,5-dimethoxytoluene (4-methyl-syringol), acetovanillone, and 2-methyl-3-heptanone were purchased from Sigma Aldrich (St. Louis, MO, USA), with purity higher than 99%. Dichloromethane (99.9%) was obtained from Thermo Fisher Scientific (Waltham, MA, USA). Alkane ladder (C7–C30) was purchased from Agilent Technologies (Santa Clara, CA, USA).

### 3.3. Preparation of Condensed Smoke Samples for Chemical Analysis

Condensed smoke (0.250 g) was diluted into 10 mL of dichloromethane and sonicated in a GT sonic (40 KHz, 25 °C, 30 min) until a clear amber-colored solution was formed. This solution was then directly transferred into 2 mL amber glass GC vials and stored at −80 °C until analysis.

### 3.4. Gas Chromatography/Mass Spectrometry–Olfactometry (GC/MS-O)

Volatile analyses were carried out on an Agilent 6890N gas chromatograph system (Agilent Technologies, Santa Clara, CA, USA) coupled with an Agilent 5973 series mass spectrometer detector (MSD) (Agilent Technologies, Santa Clara, CA, USA) and an olfactometry detection port (ODP 2) (Gerstel, Linthicum Heights, MD, USA). Helium was used as a carrier gas and held at a constant flow of 1.6 mL/min, and the effluent was split 1:1 after the GC column between the MSD and sniffing port using a capillary splitter and two deactivated fused silica columns (1 m × 0.1 mm i.d. for MS and 1 m × 0.15 mm i.d. for ODP). The ODP and MS transfer line heaters were held at 250 °C. One uL of the condensed smoke sample was injected into a 250 °C injector within a 1:10 split ratio. The GC was equipped with a DB-5MS capillary column (30 m × 0.25 mm i.d. × 0.25 μm) (Agilent, Santa Clara, CA, USA) or a DB-WAX column (60 m × 0.25 mm i.d. × 0.25 μm) (Agilent, Santa Clara, CA, USA). For the DB-5 column, the oven temperature program was as follows: the initial oven temperature was set at 40 °C for 2 min and then ramped to 250 °C (4 °C/min) and held for 8 min. For the DB-Wax column, the oven temperature was set at 40 °C for 2 min and then ramped to 180 °C (5 °C/min), ramped to 225 °C (3 °C/min), ramped to 250 °C (10 °C/min), and held for 5 min. Mass spectra were recorded at 70 eV ionization energy. The MS source temperature was set at 280 °C, the mass range was 30–350 amu, and the MS quadrupole temperature was held at 150 °C. The identification of the odor-active compounds was confirmed by comparing the mass fragmentation patterns, linear retention indices (LRI) in the two columns, and odor descriptors with those exhibited by authentic standards. The LRI values for the compounds were calculated from the retention times of n-alkanes from a C6 to C30 mixture as an external reference.

### 3.5. Aroma Extract Dilution Analysis (AEDA)

The condensed smoke was serially diluted by half-volume in dichloromethane until no further aromas were detected by GC-O to perform the AEDA. Each dilution was subjected to GC-O analysis by four experienced panelists. The panelists were familiarized with the smoke sample aromas and attribute definitions in a preparatory training session. A DB-5 MS column (30 m × 0.25 mm i.d. × 0.25 μm) was used according to the parameters listed in Section 3.4. The FD factors were determined as the last dilution at which at least two assessors were able to detect the odorant in both replicates.

### 3.6. Quantification of Odor-Active Compounds

The quantification of all odorants with FD ≥ 4 was achieved by a standard addition curve. Condensed smoke was diluted by adding 0.250 g to 10 mL of dichloromethane spiked with authentic standards for each of the analytes, ranging from 1 to 5 times the approximate concentration. The internal standard 2-methyl-3-heptanone (1 μL, 10 mg/L) was added to each sample. Each concentration level was analyzed in triplicate. Samples were held at 4 °C until analysis. One μL of each sample was injected into an Agilent 7890B GC system with Agilent MassHunter Workstation B.07.05 Software (Agilent Technologies, Santa Clara, CA, USA) equipped with a DB-5MS or DB-WAX column using a split/splitless injector set to split 10:1. The inlet temperature was set to 250 °C. Helium gas was used as a carrier with a flow rate of 1.2 mL/min. To accommodate differences in compound concentration ranges and avoid saturation at the detector, the samples were diluted from 1:10 to 1:100 in dichloromethane before injection.

For the DB-5 MS capillary column (60 m × 0.25 mm i.d.; 0.25 μm film thickness, Agilent, Santa Clara, CA, USA), the temperature program was as follows: the initial oven temperature was set at 40 °C and then ramped to 70 °C (3 °C/min), 120 °C (5 °C/min), and 300 °C (10 °C/min) and held for 4 min, and the capillary transfer line to the MSD was set to 300 °C. The DB-WAX capillary column (60 m × 0.25 mm i.d.; 0.25 μm film thickness; Agilent, Santa Clara, CA, USA) temperature program was as follows: the oven temperature was set at 40 °C and then ramped to 180 °C (5 °C/min), 225 °C (3 °C/min), and 250 °C (10 °C/min) and held for 5 min, and the capillary transfer line to the MSD was set to 250 °C.

The MS was operated in MRM mode (Agilent 7010B GC-QQQ). The quadrupole temperatures were set at 150 °C and the source temperature was 250 °C. MRM methods were optimized by the injection of pure standards by the following procedure. Each standard was first analyzed in scan mode (30–350 *m*/*z*) to identify precursor ions. Then, each precursor ion was fragmented in the second quadrupole using variable collision energies (5, 10, 15, 20, and 25 eV). Optimal product ions and collision energies were selected based on abundance and selectivity for each standard (Table 3). In the case of 2,3-butanedione, a single-ion monitoring method was used. Compounds were quantified using a 5-point standard addition curve in triplicate (*r*^2^ > 0.97), in which the negative of the x-intercept represented the compound concentration in the non-spiked sample.

### 3.7. Odor Threshold Determination for the Selected Aroma Compounds

Sensory odor threshold values for 2-methyl-2-cyclopentenone, acetoxyacetone, 3-methyl-2-(5H)-furanone, and 4-methyl-syringol were determined experimentally using a forced-choice ascending concentration series method of limits from ASTM E679-19 [53]. Fifteen panelists (ages 22–45, 5 males, 10 females) were recruited from the Ohio State University Department of Food Science and Technology (IRB # 2021E0700). The panelists evaluated a series of triangle tests that contained the compound of interest in water alongside two distilled water blanks. Five mL of each sample or water was presented in 60 mL amber bottles, which were prepared no more than 24 h before the assessments and then stored at 4 °C. Two hours before the analysis, the bottles were placed at room temperature to equilibrate. The compound was presented in ascending order; seven dilutions were assessed for each series. The standards were diluted such that, in the final set, the sample aroma was obvious to all panelists, and the first set was indiscernible. The highest concentrations of the standards were 33 μg/g for 4-methyl-syringol, 100 μg/g for 2-methyl-2(5H)-furanone, 33 μg/g for acetoxyacetone, and 300 μg/g for 2-methyl-2-cyclopentenone. The samples underwent serial dilution by a factor of 3 (1:3, 1:9, 1:27, … 1:2187). The panelists were instructed to smell each sample set, identify the different samples, and comment on any aroma perceived. All samples were evaluated in duplicate over two sessions during the same day.

The best estimate threshold (BET) for each panelist was calculated as the geometric mean of the concentration of the last incorrect set and the subsequent correct set. The overall BET was reported as the geometric mean of all panelists’ individual BETs.

### 3.8. Sensory Descriptive Analysis

The descriptive analysis was completed by an external panel at Kerry Ingredient (Beloit, WI), with over 1000 h of sensory evaluation experience and over 100 h focused on condensed smoke and smoked products (ages 28–65, 1 male and 6 females). In preparation for the analysis, the panel had previously established a lexicon for smoked products based on the work of Jaffe et al. [54]. This lexicon was then refined during three separate three-hour training sessions to identify the most relevant descriptors (8) for the evaluation of condensed smoke samples (see Table 4). The lexicon was validated via a correlation matrix to ensure no overlap between the attributes in XLSTAT (Addinsoft, Paris, France).

A recombinant model was created by dissolving authentic standards in dichloromethane at the concentrations reported in Table 1. The condensed smoke sample was prepared as described in Section 3.3. Both the condensed smoke and the recombinant samples were diluted to equal concentrations for the sensory evaluation, which was 1:40 of the original condensed smoke.

Twenty uL of each sample was portioned on a paper aroma strip, which was subsequently air-dried for 15 s to remove any residual solvent before placement in a 50 mL amber bottle. Samples were kept in the dark at 4 °C for less than 24 h and then equilibrated at room temperature for 20 min before evaluation. Samples were labeled with three-digit codes and the serving order was randomized. All references were available to the panelists during the evaluation. The panelists rated the intensity of each attribute using a 15-point scale anchored with 1 as “just recognizable” and 15 as “extremely intense”. The samples were evaluated in duplicate over two days. Responses were recorded using EyeQuestion (Logic8 B.V., Elst, The Netherlands).

The panelist performance and data analysis were evaluated using a linear regression model to investigate the three independent variables (samples, panelists, and replicates), as well as interactions for each attribute (sample*panelist, sample*replicate, and panelist*replicate), using the IBM SPSS Statistics program version 28 (IBM, Armonk, NY, USA). 

## Figures and Tables

**Table 2:** Panelist main effects and interaction terms.

		Ashy	Burnt–Sulfurous	Creosote	Green–Woody	Pungent	Smoky	Spicy–Sweet	Woody	Overall
*p*-values	Sample	0.088	0.917	0.083	0.213	0.308	0.478	0.842	0.207	0.638
	Replicate	0.697	0.086	0.083	0.955	0.269	0.666	0.434	0.293	0.336
	Sample by Panelist	0.603	0.855	0.083	0.414	0.218	0.798	0.253	0.909	0.506
	Panelist by Rep	0.199	0.368	0.754	0.955	0.897	0.885	1.000	0.293	0.958

N = 7 in duplicate. Statistics are based on a linear discrimination model incorporating the sample, the replicate, and the panelist as main effects as well as interactions. Significance was assessed at α = 0.05.

**Table 3:** Optimized MRM parameters of aroma-active compounds identified in condensed smoke.

Compound	Precursor Ion (*m*/*z*)	Product Ion (*m*/*z*)		CE (eV)
		Quantifier	Qualifier	
2,3-Butanedione *	-	86	43	-
2,3-Pentanedione	100	57	43	5
Acetol	74	43	-	5
2-Methyl-2-cyclopentenone	96	67	53	5
Acetic acid	60	45	43	10
Acetoxyacetone	86	43	-	5
Furfural	96	39	67	25
2-Acetylfuran	110	95	39	5
Butyric acid	73	55	45	5
3-Methyl-2(5H)-furanone	98	69	41	5
Guaiacol	124	109	81	15
2,6-Dimethylphenol	122	107	77	15
4-Methylguaiacol	138	123	95	15
Maltol	126	71	97	25
o-Cresol	108	107	79	15
4-Ethylguaiacol	152	137	122	15
2-Ethylphenol	107	77	79	15
2,5-Dimethylphenol	122	107	77	15
2,4-Dimethylphenol	122	107	77	15
p-Cresol	108	107	79	15
m-Cresol	108	107	79	15
4-Ethylphenol	107	77	79	15
3-Ethylphenol	107	77	79	15
3,4-Dimethylphenol	122	107	77	15
Syringol	154	139	111	15
4-Methyl-syringol	168	153	125	5
Acetovanillone	166	151	123	15

* 2,3-Butanedione was quantified in single-ion monitoring (SIM) mode.

**Table 4:** Aroma attributes with definitions and references used for descriptive analysis of condensed smoke samples.

Attribute	Definition	Reference
Ashy	The aromatics associated with the residual of burnt products and dirty ashtrays.	Ghirardelli 100% cocoa
Burnt–sulfurous	The dark, heavy, slightly sharp, and pungent notes of burning, skunk, or rubber or with the charring or burning of food.	Starbucks dark roast whole bean coffee, espresso roast, 100% arabica
Creosote	Tarry and phenolic aroma associated with smoke and solvents.	Medicasp coal tar gel shampoo
Green–woody	The aromatics associated with green wood, unseasoned wood, young branches, or saplings.	Grape stems, red seedless table grapes
Pungent	A strong, penetrating aroma or flavor resulting in a physically penetrating sensation in the nasal cavity.	Nakano rice vinegar
Smoky	Mellow and well-balanced, hardwood smoke notes.	McCormick Grill Mates mesquite seasoning
Spicy–sweet	Brown spice or sweet spice; sweet, brown, such as clove, cinnamon, nutmeg, and allspice; baking spices.	Tones ground allspice
Woody	Wood notes characteristic of bark, pits, seeds, or trees.	Great Value chopped walnuts
Overall	Intensity of overall aroma from the sample.	None

## Data Availability

Data are contained within the article.

## References

[1] Maga J.A. (1987). The Flavor Chemistry of Wood Smoke. Food Rev. Int..

[2] Jakab E. (2015). Analytical Techniques as a Tool to Understand the Reaction Mechanism. Recent Advances in Thermo-Chemical Conversion of Biomass.

[3] Paravisini L., Gourrat-Pernin K., Gouttefangeas C., Moretton C., Nigay H., Dacremont C., Guichard E. (2012). Identification of Compounds Responsible for the Odorant Properties of Aromatic Caramel: Identification of the Odorant Compounds in Aromatic Caramel. Flavour Fragr. J..

[4] Buttery R.G., Orts W.J., Takeoka G.R., Nam Y. (1999). Volatile Flavor Components of Rice Cakes. J. Agric. Food Chem..

[5] Kim S.P., Kang M.Y., Park J.C., Nam S.H., Friedman M. (2012). Rice Hull Smoke Extract Inactivates Salmonella Typhimurium in Laboratory Media and Protects Infected Mice against Mortality. J. Food Sci..

[6] Kawamoto H. (2017). Lignin Pyrolysis Reactions. J. Wood Sci..

[7] Kawamoto H., Murayama M., Saka S. (2003). Pyrolysis Behavior of Levoglucosan as an Intermediate in Cellulose Pyrolysis: Polymerization into Polysaccharide as a Key Reaction to Carbonized Product Formation. J. Wood Sci..

[8] Guillen M.D., Ibargoitia M.L. (1999). GC/MS Analysis of Lignin Monomers, Dimers and Trimers in Liquid Smoke Flavourings. J. Sci. Food Agric..

[9] Simon R., de la Calle B., Palme S., Meier D., Anklam E. (2005). Composition and Analysis of Liquid Smoke Flavouring Primary Products. J. Sep. Sci..

[10] Giri A., Zelinkova Z., Wenzl T. (2017). Experimental Design-Based Isotope-Dilution SPME-GC/MS Method Development for the Analysis of Smoke Flavouring Products. Food Addit. Contam. Part A.

[11] Guillén M.D., Ibargoitia M.L. (1999). Influence of the Moisture Content on the Composition of the Liquid Smoke Produced in the Pyrolysis Process of *Fagus sylvatica* L. Wood. J. Agric. Food Chem..

[12] Cardinal M., Cornet J., Serot T., Baron R. (2006). Effects of the Smoking Process on Odour Characteristics of Smoked Herring (*Clupea harengus*) and Relationships with Phenolic Compound Content. Food Chem..

[13] Maga J.A. (1988). Smoke in Food Processing.

[14] Guillen M.D., Manzanos M.J., Zabala L. (1995). Study of a Commercial Liquid Smoke Flavoring by Means of Gas Chromatography/Mass Spectrometry and Fourier Transform Infrared Spectroscopy. J. Agric. Food Chem..

[15] Montazeri N., Oliveira A.C.M., Himelbloom B.H., Leigh M.B., Crapo C.A. (2013). Chemical Characterization of Commercial Liquid Smoke Products. Food Sci. Nutr..

[16] Kosowska M., Majcher M.A., Jeleń H.H., Fortuna T. (2018). Key Aroma Compounds in Smoked Cooked Loin. J. Agric. Food Chem..

[17] Söllner K., Schieberle P. (2009). Decoding the Key Aroma Compounds of a Hungarian-Type Salami by Molecular Sensory Science Approaches. J. Agric. Food Chem..

[18] Majcher M.A., Jeleń H.H. (2011). Key Odorants of Oscypek, a Traditional Polish Ewe’s Milk Cheese. J. Agric. Food Chem..

[19] Pu D., Zhang Y., Zhang H., Sun B., Ren F., Chen H., Tang Y. (2020). Characterization of the Key Aroma Compounds in Traditional Hunan Smoke-Cured Pork Leg (Larou, THSL) by Aroma Extract Dilution Analysis (AEDA), Odor Activity Value (OAV), and Sensory Evaluation Experiments. Foods.

[20] Guillén M.D., Ibargoitia M.L. (1998). New Components with Potential Antioxidant and Organoleptic Properties, Detected for the First Time in Liquid Smoke Flavoring Preparations. J. Agric. Food Chem..

[21] Guillén M.D., Manzanos M.J., Ibargoitia M.L. (2001). Carbohydrate and Nitrogenated Compounds in Liquid Smoke Flavorings. J. Agric. Food Chem..

[22] Poisson L., Schieberle P. (2008). Characterization of the Key Aroma Compounds in an American Bourbon Whisky by Quantitative Measurements, Aroma Recombination, and Omission Studies. J. Agric. Food Chem..

[23] Shigeto A., Hachisuka S., Kumazawa K. (2016). Characterization of Potent Odorants in Three Different Cultivars (Madagascar, Comoro and Tahiti) of Vanilla Bean by Aroma Extract Dilution Analysis (AEDA). Food Sci. Technol. Res..

[24] Gu X., Ma X., Li L., Liu C., Cheng K., Li Z. (2013). Pyrolysis of Poplar Wood Sawdust by TG-FTIR and Py-GC/MS. J. Anal. Appl. Pyrolysis.

[25] Rychlik M., Schieberle P., Grosch W. (1998). Compilation of Odor Thresholds, Odor Qualities and Retention Indices of Key Food Odorants.

[26] Leffingwell, J.C.; Leffingwell, D.. http://www.leffingwell.com/odorthre.htm.

[27] Fenaroli G., Burdock G.A. (2010). Fenaroli’s Handbook of Flavor Ingredients.

[28] Van Gemert L.J. (2011). Flavour thresholds: Compilations of Flavour Threshold Values in Water and Other Media.

[29] Pang X., Yu W., Cao C., Yuan X., Qiu J., Kong F., Wu J. (2019). Comparison of potent odorants in raw and ripened pu-erh tea infusions based on odor activity value calculation and multivariate analysis: Understanding the role of pile fermentation. J. Agric. Food Chem..

[30] Czerny M., Christlbauer M., Christlbauer M., Fischer A., Granvogl M., Hammer M., Hartl C., Hernandez N.M., Schieberle P. (2008). Re-investigation on odour thresholds of key food aroma compounds and development of an aroma language based on odour qualities of defined aqueous odorant solutions. Eur. Food Res. Technol..

[31] Marcq P., Schieberle P. (2015). Characterization of the key aroma compounds in a commercial amontillado sherry wine by means of the sensomics approach. J. Agric. Food Chem..

[32] EFSA Panel on Food Contact Materials, Enzymes, Flavourings and Processing Aids (CEF) (2010). Scientific Opinion on Safety of Smoke Flavour—Primary Product–AM 01. EFSA J..

[33] EFSA Panel on Food Contact Materials, Enzymes, Flavourings and Processing Aids (CEF) (2010). Scientific Opinion on the Safety of Smoke Flavour Primary Product Scansmoke R909. EFSA J..

[34] EFSA Panel on Food Contact Materials, Enzymes, Flavourings and Processing Aids (CEF) (2010). Scientific Opinion on Safety of Smoke Flavour Primary Product—TRADISMOKE^TM^ A MAX. EFSA J..

[35] EFSA Panel on Food Contact Materials, Enzymes, Flavourings and Processing Aids (CEF) (2011). Scientific Opinion on the Safety of Smoke Flavour Primary Product Zesti Smoke Code 10-2011 Update. EFSA J..

[36] European Food Safety Authority (EFSA) (2009). Safety of Smoke Flavour Primary Product—Scansmoke PB 1110. EFSA J..

[37] European Food Safety Authority (EFSA) (2009). Safety of Smoke Flavour Primary Product—Scansmoke SEF7525. EFSA J..

[38] European Food Safety Authority (EFSA) (2009). Safety of Smoke Flavour Primary Product Smoke Concentrate 809045. EFSA J..

[39] European Food Safety Authority (EFSA) (2009). Safety of Smoke Flavour Primary Product—SmokEz C-10. EFSA J..

[40] European Food Safety Authority (EFSA) (2009). Safety of Smoke Flavour Primary Product—SmokEz Enviro 23. EFSA J..

[41] European Food Safety Authority (EFSA) (2009). Safety of Smoke Flavour Primary Product Unismoke. EFSA J..

[42] Rowell R., Pettersen R., Tshabalala M. (2012). Cell Wall Chemistry. Handbook of Wood Chemistry and Wood Composites.

[43] Adler E. (1977). Lignin Chemistry: Past, Present and Future. Wood Sci. Technol..

[44] Fagerson I.S. (1969). Thermal Degradation of Carbohydrates; a Review. J. Agric. Food Chem..

[45] Ginz M., Balzer H.H., Bradbury A.G.W., Maier H.G. (2000). Formation of Aliphatic Acids by Carbohydrate Degradation during Roasting of Coffee. Eur. Food Res. Technol..

[46] Fricke K., Schieberle P. (2020). Characterization of the Key Aroma Compounds in a Commercial Milk Chocolate by Application of the Sensomics Approach. J. Agric. Food Chem..

[47] Grosch W. (1994). Determination of Potent Odourants in Foods by Aroma Extract Dilution Analysis (AEDA) and Calculation of Odour Activity Values (OAVs). Flavour Fragr. J..

[48] Akiyama M., Murakami K., Hirano Y., Ikeda M., Iwatsuki K., Wada A., Tokuno K., Onishi M., Iwabuchi H. (2008). Characterization of Headspace Aroma Compounds of Freshly Brewed Arabica Coffees and Studies on a Characteristic Aroma Compound of Ethiopian Coffee. J. Food Sci..

[49] Grosch W. (1998). Flavour of Coffee. A Review. Nahrung.

[50] Curioni P.M.G., Bosset J.O. (2002). Key Odorants in Various Cheese Types as Determined by Gas Chromatography-Olfactometry. Int. Dairy J..

[51] Wang H., Chambers E. (2018). Sensory Characteristics of Various Concentrations of Phenolic Compounds Potentially Associated with Smoked Aroma in Foods. Molecules.

[52] Wang H., Chambers E., Kan J. (2018). Sensory Characteristics of Combinations of Phenolic Compounds Potentially Associated with Smoked Aroma in Foods. Molecules.

[53] E18 Committee (2019). Practice for Determination of Odor and Taste Thresholds By a Forced-Choice Ascending Concentration Series Method of Limits.

[54] Jaffe T.R., Wang H., Chambers E. (2017). Determination of a Lexicon for the Sensory Flavor Attributes of Smoked Food Products. J. Sens. Stud..

